# Revascularization of acute stent thrombosis caused by diarrhea after carotid artery stenting in an intermediate clopidogrel metabolizer

**DOI:** 10.1186/s12883-023-03293-5

**Published:** 2023-07-06

**Authors:** Huaishun Wang, Longdong Xu, Yan Qin, Guodong Xiao

**Affiliations:** 1 Second Affiliated Hospital of Soochow University, Suzhou, 215004 China; 2 fifth People’s Hospital of Changshu, Suzhou, 215004 China; 3 Department of Neurology and Suzhou Clinical Research Center of Neurological Disease, Second Affiliated Hospital of Soochow University, No. 1055 Sanxiang Road, Suzhou, 215004 China

**Keywords:** Carotid artery stenting, Acute stent thrombosis, Thrombectomy

## Abstract

Carotid artery stenting (CAS) is an alternative treatment to carotid endarterectomy for carotid artery stenosis. Acute stent thrombosis (ACST) is an extremely rare complication but can have devastating consequences. Although many cases have been reported, the best treatment is still uncertain. In this study, we report the treatment of ACST caused by diarrhea in an intermediate clopidogrel metabolizer. We also review the literature and discuss appropriate treatment strategies for this rare event.

## Background

The ACAS (Asymptomatic Carotid Atherosclerosis Study) and the NASCET (North American Symptomatic Carotid Endarterectomy Trial) demonstrated carotid endarterectomy (CEA) is superior to medical therapy alone. [1, 2] Over the past few decades, with advances in device technology, carotid artery stenting (CAS) has become an alternative treatment for carotid artery stenosis, which was noninferior to CEA. [3, 4] Acute stent thrombosis (ACST) is an extremely rare complication after CAS with an incidence of 0.5 to 0.8%. [5] However, it can have devastating consequences. [6] The etiology of ACST is complex and the treatment method is not clear at present.

In this study, we report the treatment of ACST caused by diarrhea in an intermediate clopidogrel metabolizer. We also review the literature and discuss appropriate treatment strategies for this rare occurrence.

## Case presentation

**Fig. 1:**
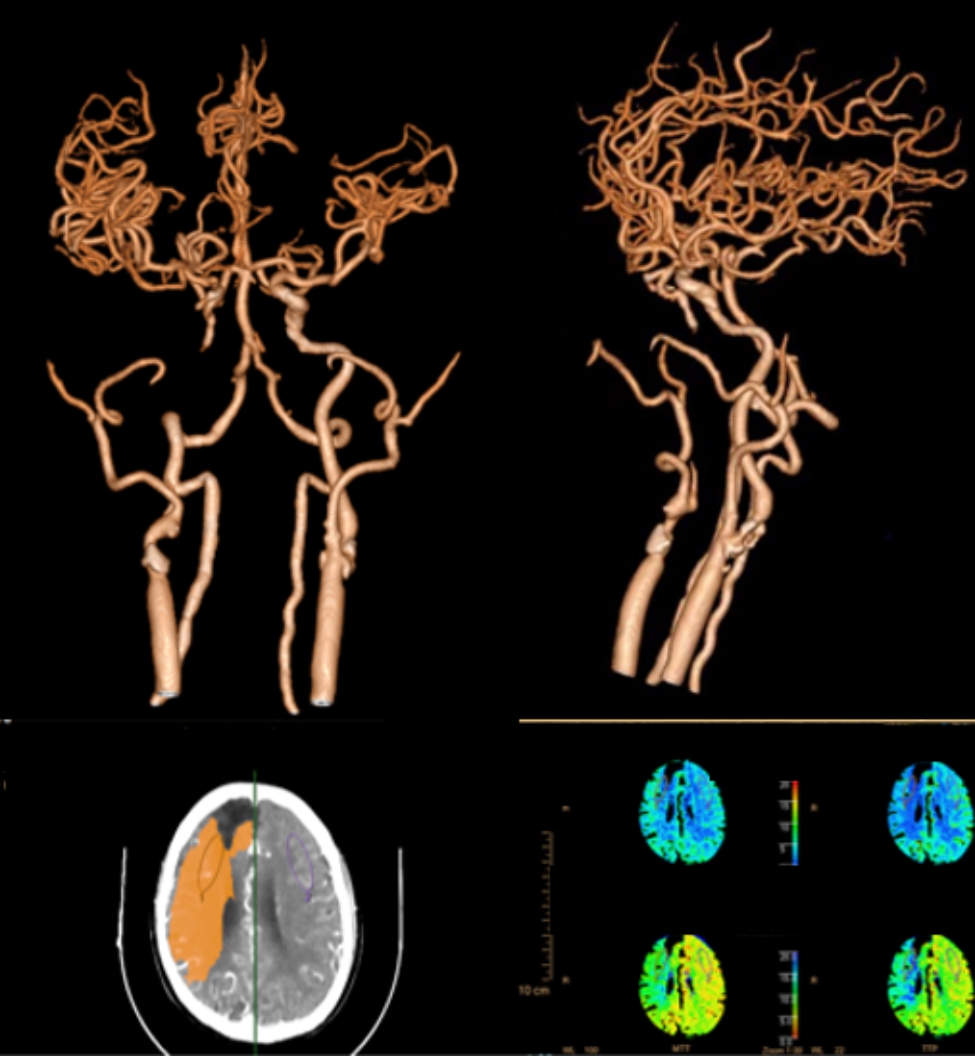
CTA and CTP three days after admission

A 74-year-old male patient requiring CAS was diagnosed with a right frontal and basal ganglia infarction 3 months ago. CT angiography (CTA) and CT perfusion (CTP) of the brain showed right internal carotid artery (ICA) occlusion with a hypoperfusion in the right cerebral hemisphere and left ICA severe stenosis (Fig. 1). The patient had a history of hypertension and had been treated with nifedipine for a long time. Blood pressure was controlled well. His nervous system physical examination was normal at admission. No obvious abnormalities were found in the patient’s laboratory examination, electrocardiogram or cardiac ultrasound. Aspirin (100 mg) combined with clopidogrel (75 mg) were given before CAS. It should be noted that the patient had diarrhea for 3 consecutive days before CAS with 6 bowel movements per day. Antidiarrheal treatment was given.

**Fig. 2:**
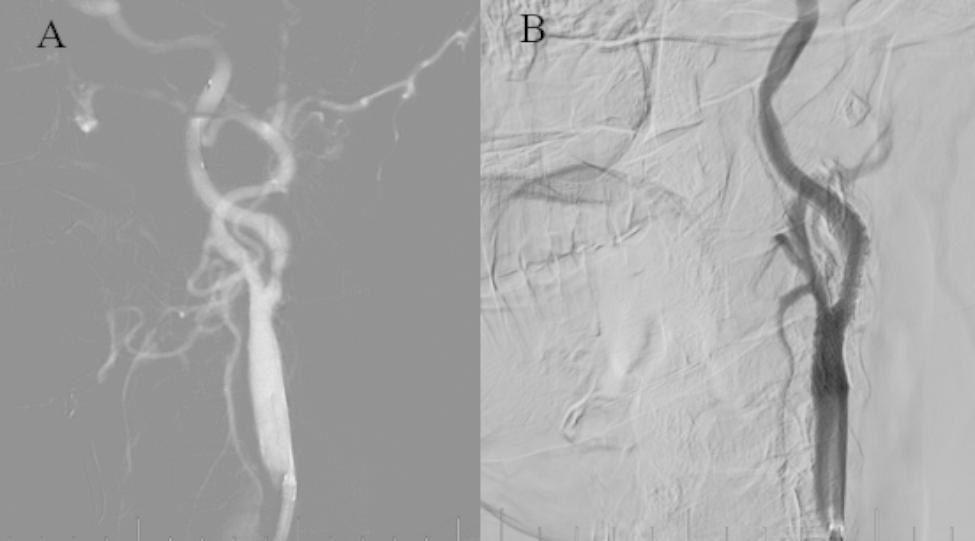
**A** shows severe stenosis of the left ICA. **B** shows the left ICA after CAS.

Our plan was to perform CAS on left ICA with severe stenosis. The Spider 5 mm distal embolic protection device was placed in the far end of the C1 segment of ICA. INVATEC 4*30 mm was used to dilate the stenosis and Precise 9*40 mm was implanted (Fig. 2). On the first day after CAS, the patient’s genetic test showed that the patient had an intermediate metabolic of clopidogrel. We adjusted the treatment to include aspirin that was combined with cilostazol.

**Fig. 3:**
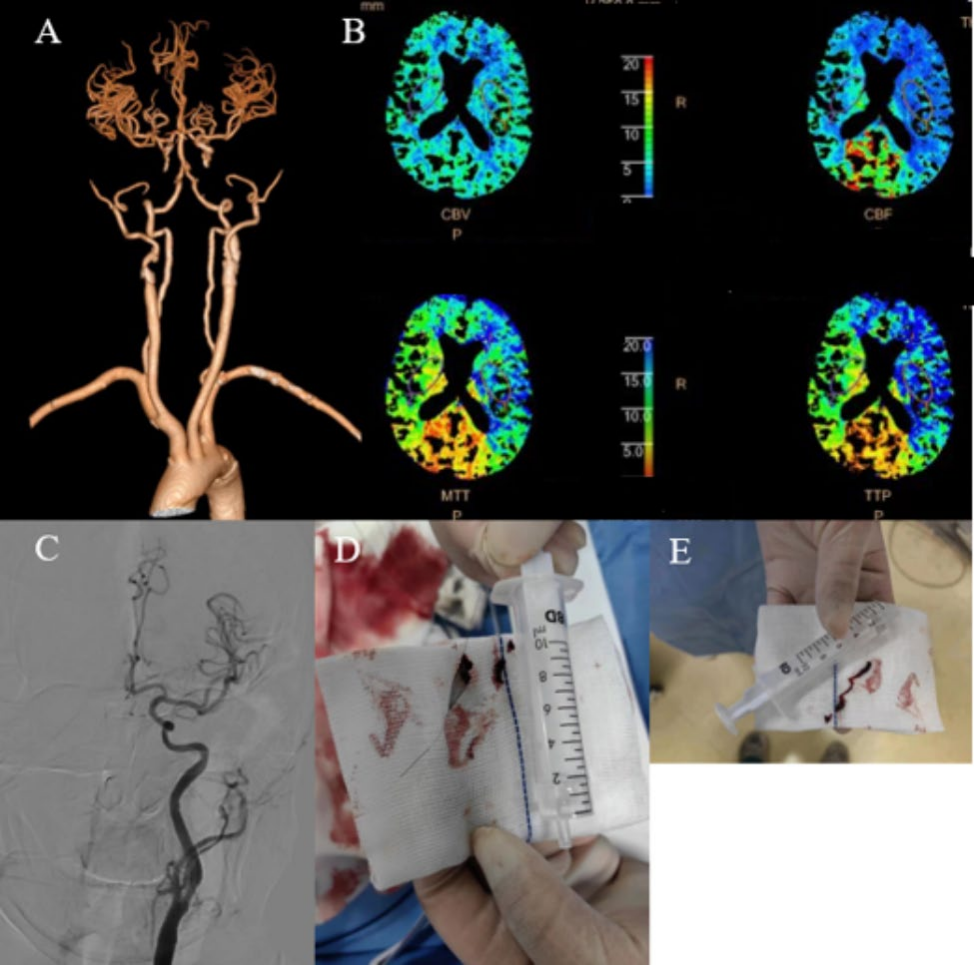
**A** shows the patient’s CTA after symptom aggravation. **B** shows the patient’s CTP after symptom aggravation. **C** shows DSA of the patient after thrombectomy. **D-E** shows the thrombus after thrombectomy

On the morning of the second day after CAS, the patient experienced sudden speech failure accompanied by right limb weakness after transitioning from standing to decubitus. We did a rapid CTA and CTP, which revealed ACST in the left ICA. Subsequently, thrombectomy was performed on the patient. Spider 5 mm embolic protection was placed at the distal end of the C1 segment. We performed a successful aspiration thrombectomy by using an aspiration catheter (Fig. 3). Eventually, the patient’s neurological function fully recovered without any residual effects.

## Discussion

ACST is a rare complication of CAS, occurring in less than 1% of patients. The causes of ACST include insufficient antiplatelet therapy, early discontinuation of antiplatelet therapy, clopidogrel resistance, hypercoagulability, soft plaque and its protrusion, local vascular dissection, vasospasm, and intimal injury. [7–9] We tested the CYP2C19 status of our patient, which was CYP2C19*1/*2. We adjusted the patient’s medication from clopidogrel to cilostazol on the first day after CAS. It should be noted that the patient had diarrhea from 3 days before CAS until the day of CAS. No association between diarrhea and acute thrombosis has been previously reported. We believed that this may be caused by hypercoagulability caused by dehydration. Some studies have indicated that hemoconcentration following dehydration contributes to an increase in pivotal factors involved in blood clotting cascades, such as platelets and fibrinogen. [10] A experimental study showed dehydrated mice fed for 9 days triggered an increase in von Willebrand factor (vWF) level, which subsequently assisted in clotting activation. [11] In addition, postural changes and massive loss of blood volume due to diarrhea may be the cause of ASCT in the patient.

ACST is a rare complication for which the best treatment is unknown. The patient has acute onset and severe symptoms. We immediately performed thrombus aspiration, which was conducted under the protection of distal embolic protective devices in order to prevent thrombus detachment from causing middle cerebral artery occlusion. Antiplatelet, anticoagulant, thrombolysis, thromboendarterectomy, mechanical thrombectomy and aspiration were all reported. [8, 12–15] Rapid revascularization with more aggressive treatment may be justified in patients with severe disease deterioration. [14] Treatment for patients with asymptomatic or mild neurological impairment remains unclear.

Both dehydration and clopidogrel resistance were risk factors for ACST in the patient. The cause of ACST might be caused by their combined action. Perioperative management needs attention to reevaluated. Not only the treatment of doctors, but also the cooperation of the nursing team is crucial.

## Data Availability

Data sharing is not applicable to this article as no datasets were generated or analysed during the current study.
